# Fueling Inner Resources Through Co-Creation: A Scoping Review on the Impact of Co-Creation of Education on Learners’ Well-Being

**DOI:** 10.5334/pme.1726

**Published:** 2025-03-25

**Authors:** Shireen Suliman, Muhammad Zafar Iqbal, Abdel Hakim Bishawi, Margaret Allen, Karen D. Könings

**Affiliations:** 1 Hamad Medical Corporation, Doha, Qatar; 2 College of Medicine, QU Health, Qatar University, Doha, Qatar; 3 Weill Cornell Medicine Qatar, Qatar; 4 School of Health Professions Education, Maastricht University, Maastricht, The Netherlands; 5 Research Department, Acuity Insights, Toronto, Ontario, Canada; 6 QU Library, Qatar University, Doha, Qatar; 7 Medical Education Department, Hamad Medical Corporation, Doha, Qatar; 8 School of Health Sciences, University of East Anglia, Norwich, UK

## Abstract

**Introduction:**

Co-creation is gaining momentum in health professions education with positive effects on learners’ engagement and motivation. Concurrently, emotional challenges faced by learners—such as stress, anxiety, burnout, and depression—continue to be a problem for health professions education leaders. This review seeks to explore how learners’ active participation in shaping their educational experience may influence their well-being.

**Methods:**

We searched MEDLINE, Scopus, Web of Science, and CINHAL. We included studies conducted within a health professions education context, which involved learners in curriculum design processes, and reported outcomes related to learners’ well-being. We used Seligman’s PERMA model to report these terms.

**Results:**

Of 4222 reviewed articles, 24 met the inclusion criteria. All studies reported outcomes related to learners’ well-being across the five domains of the PERMA model: Positive Emotions (n = 23), Meaning (n = 20), Positive Relationships (n = 10), Engagement (n = 4), and Accomplishment (n = 4). Studies describing a true student-staff partnership approach (n = 17) involved smaller learner groups, focused on developing new curricula reported outcomes related to learners’ positive well-being in all five domains. Studies describing pseudo-partnership (N = 7) also reported positive learner well-being, but not in all domains, and mostly focused in the context of developing extracurricular mental health initiatives.

**Conclusions:**

Co-creation, especially when true student-staff partnership is used, can have a positive influence on learners’ well-being. This underscores the importance of empowering learners to participate in shaping their education. Lessons learned from this review may encourage curriculum planners and education leaders to create opportunities and initiatives for involving learners in the design of their education.

## Introduction

Academia faces a growing challenge in addressing emotional issues such as stress, anxiety, burnout, and depression experienced by learners during their health professions education (HPE). A way to surmount these emotional challenges could be to actively engage learners, including them as partners in designing their education so that they feel internally driven, empowered, and autonomous. This close collaboration between learners and teachers, commonly known as co-creation [1], aims to improve teaching and learning by incorporating learners’ perspectives in the educational design process [2]. This approach gives students more control over their learning and allows them to voice their opinions during curricular reforms to ensure their unique needs and interests are addressed [1,3,4]. Research has shown that co-creation enhances learners’ motivation, empowerment, and sense of belonging by fostering a secure and safe collaborative environment [5,6,7]. Co-creation also supports the development of positive relationships and a stronger academic community [8]. Learner involvement is now seen as a way to address various educational challenges [9]; however, its influence on learners’ well-being in the context of (often stressful) HPE has not been documented yet [10,11].

Well-being is a state of positive feelings and meeting one’s full potential in the world [12]. Seligman presents the PERMA model, which outlines five essential components of well-being and happiness: (1) Positive Emotions: Experiencing feelings like joy and contentment; (2) Engagement: Feeling absorbed and actively involved in activities; (3) Positive Relationships: Feeling supported and respected by others; (4) Meaning: Believing life is valuable and feeling connected to something greater; (5) Accomplishment: Progressing toward goals and experiencing a sense of achievement [13]. This model facilitates understanding the elements that enhance life satisfaction and creativity [13]. In health professions education, experiencing positive emotions (such as enjoyment, happiness, relief, or pride) during training is important as it unlocks human cognition and encourages learners to think more freely, thoughtfully, and creatively, transposing positive mental and psychological health outcomes [14]. Positive emotions lead to more enduring effects, serving as catalysts for personal development and social bonds. By building people’s personal and social resources, positive emotions transform people for the better, giving them better lives in the future [14]. Learners can then widen the range of potential coping strategies during unfavorable circumstances that can, in return, enhance their resilience against present and future adversities [15,16,17]. Studies examining the PERMA domains reveal protection against stress [18], reduction in depressive symptoms, and decreased job burnout [19,20]. Therefore, we aim to use the PERMA-model from positive psychology as a lens to explore how co-creation interventions might reduce burnout and enhance well-being in highly stressful careers like HPE [20].

According to the Control-Value Theory of Achievement Emotions (CVTAE), individuals experience specific emotions depending on the extent to which they feel they have agency over achievement activities or outcomes (i.e., perceived control) and whether they consider these activities or outcomes important [21,22]. When individuals are given control over a task that they highly value, it leads to positive emotions such as enjoyment, motivation, and overall psychological well-being [21,23]. In a recent amendment to the theory, it was suggested that low-control and negative emotions like anxiety, shame, and hopelessness could harm health by disrupting stress management while predicting students’ self-reported psychosomatic health problems [24]. Therefore, involving learners to collaborate with teachers in the educational design process and giving them the autonomy to shape their learning experiences actively [25] is expected to elicit positive emotions, which may help them combat stressful circumstances during their training.

While co-creation of educational design is encouraged, it is equally important to ensure that learners contribute actively and equally to such activities. Without explicit attention to the dynamics of this partnership, learners may be identified as partners, but their role remains implicit and passive, thus leading to pseudo-partnership [9,26]. The process-outcome model of student-staff partnership defines different levels of learner involvement as partners (the process) and their inclusiveness in curriculum design (the outcome) [27]. This model indicates that some studies treat learners as data resources, relying on their feedback or survey results without recognizing their roles as partners. Although learners may be involved, their input is limited, resulting in pseudo-partnerships. In a true partnership, open dialogue allows all participants to contribute equally in various ways to curriculum and pedagogy [28]. In line with the CVTAE, a true student-staff partnership established during the co-creation process is anticipated to elicit positive emotions in learners. However, our understanding is limited in this domain as the influence of co-creation on learners’ well-being has not been comprehensively explored. Therefore, this review explores: 1) the reported outcomes related to learners’ well-being in studies where learners were involved in the design of their education, and 2) whether there are any differences in the reported outcomes related to learners’ well-being between true and pseudo-partnership studies. The results of this review study may help educators, curriculum planners, and academic leaders understand the value of co-creation and its potential influence on learners’ well-being. This understanding could then inspire them to develop initiatives and actively involve learners in those partnership activities that would positively influence learners’ well-being.

## Methods

**Figure 1:**
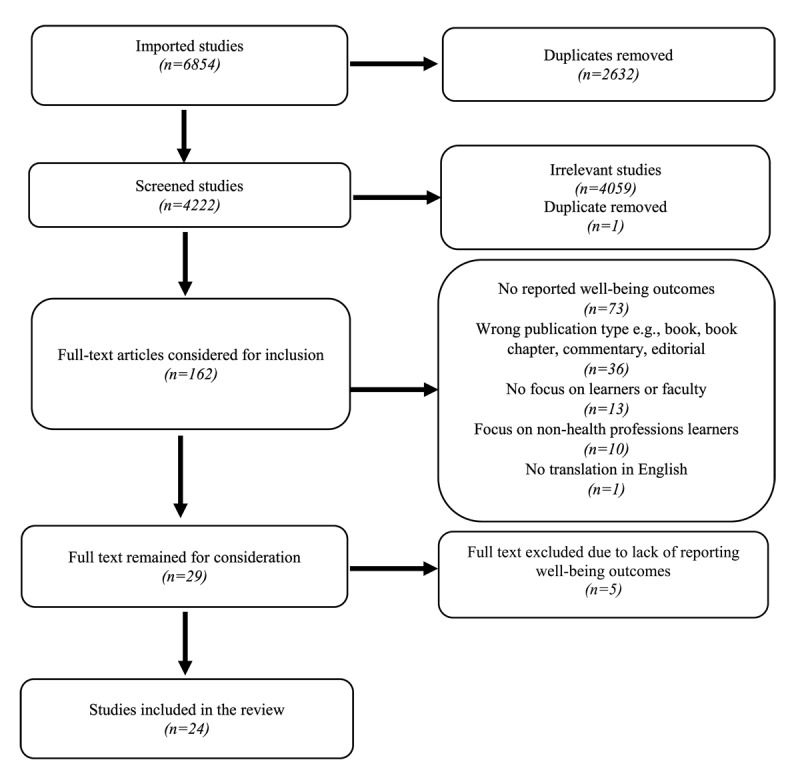
Preferred Reporting Items for Systematic Reviews and Meta-Analyses (PRISMA) flow diagram for the scoping review process [30]. This diagram outlines the steps through which the author selected the studies for inclusion in this scoping review.

We used a scoping review methodology to map the key concepts of our research systematically [29]. Following Arksey and O’Malley’s five-step framework, [29] we reported our findings using the PRISMA Extension for Scoping Reviews checklist [30] and Joanna Briggs Institute (JBI) guidelines [31]. The review protocol is shown in Figure 1.

### Step 1: Identification of a research question

Guided by the CVTAE, this scoping review is designed to map literature exploring the reporting of learners’ specific emotions based on their active participation in educational design. The review aims to answer the questions: “What are the outcomes related to learners’ well-being as reported in studies that involved learners in designing their education? Are there any differences in the reported outcomes related to learners’ well-being between true and pseudo-partnership studies?”.

### Step 2: Identifying relevant research studies

An academic health sciences librarian performed a thorough search in MEDLINE, Scopus, Web of Science, and CINHAL, focusing on English-language papers published until October 23, 2023. To cover the diverse terminology related to the co-creation concept, multiple search terms were used (See Appendix 2 for details).

### Step 3: Study selection

All articles were imported into Covidence, where two researchers, SS and MZI, independently screened 5% of the total articles by title and abstract to establish initial inclusion and exclusion criteria. Of the 300 screened articles, 41 (13.7%) resulted in conflict resolution through discussion. In a second round, 309 articles were screened, with only 11 (3.5%) leading to conflicts. In the light of the CVTAE, articles were included if: (1) learners were given control over their curriculum by participating in educational design, (2) there was reported impact on the participating learners’ well-being – not necessarily as a primary outcome of the study, (3) they reported an original research study, and (4) the population involved learners from any health profession. Articles were excluded if: (1) learners were not from health professions, (2) partnership involved patients or community rather than teaching staff, (3) no reported terms that indicate learners’ well-being, (4) they were commentaries or editorials, or (5) they were in a language other than English. After agreeing on the screening approach, one researcher (SS) screened the remaining 4,222 articles, excluding 4060. The two researchers divided the 162 shortlisted articles for full-text screening. SS reviewed 112 articles and shortlisted 21, while MZI reviewed 50 and shortlisted eight. SS then re-evaluated the 133 excluded articles and identified four that were excluded in a consensus meeting, finalizing the selection to 29. SS and MZI then reviewed each other’s shortlisted articles, resulting in conflicts over five articles that were excluded after a third consensus meeting. Ultimately, 24 articles met the criteria for analysis and reporting (see Figure 1). KK and MA reviewed and approved the final list for further analysis.

### Step 4: Charting the data

**Figure 2:**
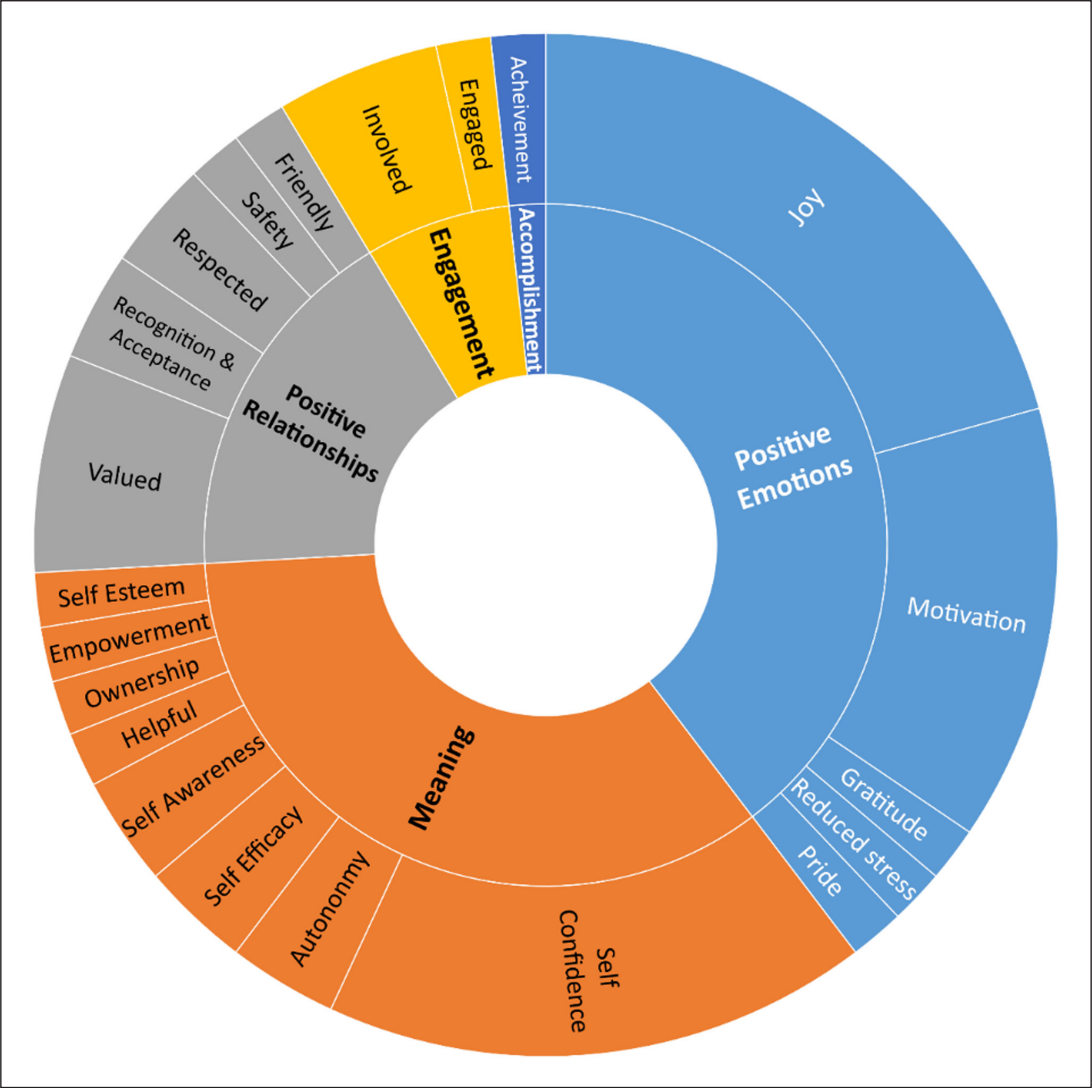
Representation of the reported outcomes related to well-being according to the PERMA model of Well-being: Dominance of the Positive Emotions and Meaning domains, followed by Positive relationships. Engagement and Accomplishment domains were less prevalent in the included studies.

Following JBI guidelines for Scoping Reviews, SS created an analytical framework in Microsoft Excel to extract information from each study. The extraction sheet, calibrated by MZI and reviewed by the research team, focused on four main areas: 1) study characteristics, 2) co-creation process, 3) co-creation focus, and 4) reported terms that indicate learners’ well-being. We documented each study’s title, author(s), publication year, journal name, and methodology. Additionally, we examined the co-creation process, considering terminology, setting, participant roles, sample size, and partnership level (true or pseudo). To distinguish between the two, we used the definition of partnership as ‘*a process of student engagement, understood as staff and students learning and working together to foster engaged student learning and engaging learning and teaching enhancement*’ (p. 7) [32]. We also applied our process-outcome model of student–staff partnership [27]. A true partnership features equal involvement from both students and staff, while a pseudo-partnership lacks active and direct student input, often incorporating their voices only indirectly through quotes or survey extrapolations [9]. SS and MZI reassessed their classification of partnership based on the co-creation setting and interactions between staff and learners. Student-staff partnership is a relational process grounded in a culture that encourages collaboration among students and staff members [33]. A lack of direct interaction between staff and students impaires this partnership, leading to only the indirect inclusion of student voices and creating a pseudo-partnership. Lastly, we charted the co-creation outcomes, exploring what was co-created, the targeted population, and whether any terms that indicate learners’ well-being were reported, as they might not necessarily be the focus of those studies. Guided by Seligman’s PERMA model of well-being [34], we analyzed and categorized the reported terms that indicate learners’ well-being and illustrated their representation in Figure 2. See Supplementary materials Table 1 and Figure 2 for more details.

### Step 5: Collating, summarizing, and reporting results

Data captured in the Excel sheet was independently summarized by SS and MZI. To illustrate the scoping review results, we followed Peters et al. [31] recommendations that suggest presenting the results in tabular and descriptive formats while linking them to the review objective(s) (See Table 1).

## Results

We present the findings of this scoping review in four subsections: an outline of the included studies, the co-creation process, the co-creation focus, and the reported outcomes that indicate learners’ well-being.

### Outline of the included studies

The key characteristics from the studies in this scoping review are summarized in the supplementary Table 1. Of the 24 included articles, 17 (71%) were published in or after 2020; nine (38%) in 2023, and seven (29%) were from 2010 to 2020. Thirteen studies were based in Europe, three in North America, three in Australia, two in Canada, one in New Zealand, one in Asia, and one across continents. Methodologically, 15 articles (63%) used qualitative methods, and nine (37%) used mixed methods. Most articles (23) focused on undergraduate learners, with only one including postgraduate learners [35]. In terms of HPE, ten studies were related to Medicine, three to Dentistry, one each to Pharmacy and Nursing, two to Population Health, one to Biomedical Sciences, and six to mixed disciplines, including allied health and STEM fields.

### The co-creation processes

This section outlines the methodology for the co-creation sessions, including participant numbers and settings. All 24 studies used qualitative group discussions to engage learners in educational design. Various terms were employed: “sessions” (co-creation, evaluation, training, planning) in six studies, “workshops” (design, co-design, co-creation) in four, “meetings” in four, and “focus group discussions” in three. One study included both “meetings and sessions,” while another featured a mixed (meetings and discussions). The sample size per group was relatively small, with 19 studies having fewer than 10 participants and five haing between 10 and 20. In 17 studies, true student-staff partnership was evident through direct interaction and collaboration between the learners and the staff. Group sizes varied from three to 16. In contrast, seven studies are classified as pseudo-partnerships where learners were involved in the design process but had no direct interaction with the staff. In these studies, learners discussed the design process and provided feedback in separate groups organized for each stakeholder group. Sample sizes in these studies ranged from three to 19 participants.

### The focus of the co-creation

Fifteen studies (63%) focused on developing curricular courses, eight on extracurricular activities, and one on mixed content. Out of fifteen studies focused on curricular content development, nine developed new curricular content, four revised existing content, and two sought learner input for both. Thirteen studies described learner involvement in developing complete curricular content, while two focused on course evaluation [36] and ongoing feedback [37]. Among eight studies that developed extracurricular activities, seven described mental health interventions [35,38,39,40,41,42,43]. Six of these studies involved learners at a pseudo-partnership level.

### The reported outcomes related to learners’ well-being

All 24 studies showed that learners involved in co-creation experienced positive well-being outcomes. Only one study reported frustration due to peer disengagement; it also reported that most students enjoyed the collaborative process [44].

Of the 24 included studies, 23 reported high Positive Emotions, 20 reported Meaning, 10 reported Positive Relationships, four reported Engagement, and only one reported Accomplishment, as illustrated in Figure 2 in color-coded domains. In the Positive Emotions domain, 50% reported feelings of *joy*, while 33% reported *motivation and enthusiasm*. Only a few studies (4%) reported *gratitude, reduced stress*, and *pride*. In the Meaning domain, *self-confidence* was reported in 42% of the studies, *autonomy, self-awareness, self-efficacy* in 8%, and *helpfulness, ownership, empowerment*, and *self-esteem* in 4% each. Positive Relationships in studies were evident through learners feeling *valued* (17%), *respected* (8%), *recognized and accepted* (8%), *friendly* (4%), and *safe* (4%). In terms of Engagement, learners reported positive feelings of *involvement* (13%) and *engagement* (4%), while only 4% reported feelings of *achievement* in the Accomplishment domain.

***Positive emotions:*** Of the 24 studies, 12 reported learners’ feeling of *joy* during co-creation, attributed to the opportunity to be involved [45] and collaborate with the faculty [46]. Learners enjoyed the feeling of responsibility and leadership and viewed the activities as highly worthwhile [37], with friendly atmosphere [41], pleasant co-creation exercises [47], room for creativity [48], and interprofessional interactions [44]. One study linked enjoyment with learners’ autonomy [39], and another study observed that learners enjoyed it when the co-created curricular changes were implemented in their semester cohort [43]. Eight studies reported that learners experienced *motivation* and *enthusiasm*, appreciating the opportunity to work with teachers and share their cases/viewpoints, making them feel more relaxed and stimulated [49]. Three studies reported learners’ feelings of *gratitude* [50], *pride* [48], and *reduced stress* when co-creating with faculty [51]. Overall, learners viewed their co-creation experience positively, gaining insights into their strengths and weaknesses, and felt like they were part of a team, which boosted their enthusiasm and confidence [39].

***Engagement:*** Three studies reported learners’ feelings of *involvement* [35,40,48], with one noting increased *engagement*, motivation, ownership, and meta-cognitive learning [40,48]. Learners felt empowered [40] by participating in co-creation and believed they could make meaningful changes in education [35].

***Positive relationships:*** Four studies found that learners felt *positively valued* through involvement in educational design [35,40,48,50]. Two studies mentioned that they felt *respected* and *inclusive*, which empowered them [40,50], while another two highlighted feelings of being *recognized and accepted* [35,52]. Co-creation workshops allowed learners to express their thoughts and frustrations openly and feel acknowledged [44,52]. One study described the co-creation environment as *friendly* [41], while another emphasized that learners felt *safe and comfortable* [53].

***Meaning:*** Ten studies reported that the co-creation process enhanced learners’ *self-confidence*, self-reflection, and peer teaching abilities [54]. When encouraged to speak up, learners were able to contribute productively to the conversations [55]. Two studies noted learners’ increased *self-awareness* [39,48] and *self-efficacy* [48,56], while one reported improved *self-esteem* [39]. Overall, involvement in co-creation *empowered* learners [40], giving them greater *autonomy* [39,56] and *ownership* [48] and highlighting their *helpfulness* in the design process [40].

***Accomplishment:*** Only in one study did learners report that being engaged in the design process helped them to be productive and accomplish outputs they felt confident in [48].

### Well-being related outcomes in relation to study characteristics

Concerning the PERMA model, all HPE studies reported on the Meaning domain. Positive Emotions were reported in all professions except for the one study that involved nursing learners [42]; Positive Relationships were highlighted in studies involving medicine and mixed professions. Engagement and Accomplishment domains were reported only in studies involving biomedical learners. The majority of studies had a small sample size. Studies (n = 19) with fewer than ten participants reported outcomes related to learners’ well-being across all five PERMA domains. Studies (n = 5) with larger groups of more than ten participants reported only on Positive Emotions and Positive Relationships. Studies involving learners in curricular and extracurricular activities demonstrated positive well-being outcomes. In studies focusing on extracurricular content (n = 8), Positive Emotions, Meaning, and Positive Relationships were observed. Studies involving learners in developing new curricula (n = 16) reported outcomes related to learners’ well-being in all five domains. However, studies revising existing curricula (n = 6) reported outcomes in only three domains (Positive Emotions, Meaning, and Positive Relationships).

### Well-being outcomes in relation to the type of partnership

**Figure 3:**
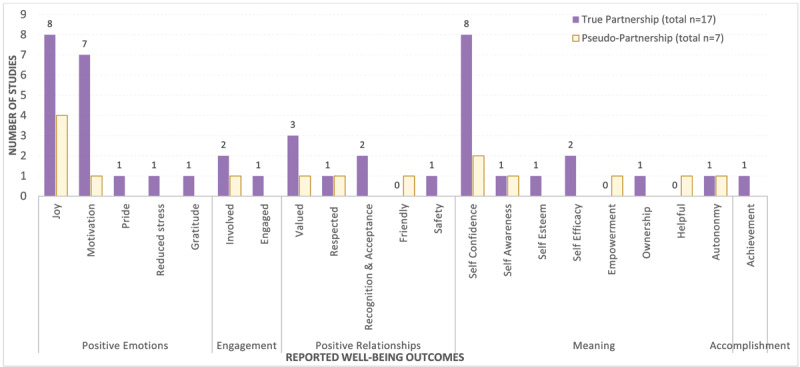
The number of studies reporting the different outcomes related to learners’ well-being, separately for studies reporting true student-staff partnership (total n = 17) and pseudo-partnership (total n = 7).

Figure 3 demonstrates reported well-being outcomes separately for true student-staff partnership studies (n = 17) and pseudo-partnership studies (n = 7). In both tyes of studies, participants mentioned feelings of *joy* and *motivation*. However, feelings of *pride, gratitude*, and *reduced stress* were only reported in true partnership studies. Both types of studies reported *self-confidence* and *self-awareness* in the domain of Meaning. However, true partnership studies only discussed *self-esteem, self-efficacy*, and *ownership*. Feeling *empowered* [55] and being *helpful* [40] were mentioned only in the studies with pseudo-partnership. Regarding the domain of Positive Relationships, both study types mentioned that learners felt *valued* and *respected* in student-staff partnerships. True partnership studies highlighted feelings of *recognition, acceptance* and *safety*, while pseudo-partnership studies emphasized *friendliness* [41]. Outcomes related to learners’ well-being were reported across four domains in both true and pseudo-partnership studies, whereas the Accomplishment domain was only reported in true partnership studies.

## Discussion

The well-being of learners remains a challenge in HPE. In this review, we – a group of scholars with interest and experience in co-creation – explored how involving learners as partners in designing their education might bring positive well-being outcomes among co-creating learners. We have seen a notable increase over time in studies using a co-creation approach within various HPEs, with the majority published in the last five years, originating from European countries and employing a qualitative methodology. The increasing citations on co-creation, as demonstrated by Könings et al. [4], reflect a growing interest in the subject, which likely explains the qualitative methodology adopted in the included studies, allowing for a deeper exploration of this emerging phenomenon [57]. We found that most studies included a small sample size of participants and used formats of learner involvement, like meetings and workshops. Larger groups often struggle to engage meaningfully in student-staff partnerships [58]. We also found most of the studies in the field of medicine and at the undergraduate level, likely due to significant curricular reforms in medical schools [59].

The included studies thoroughly covered all aspects of Seligman’s PERMA model, particularly emphasizing the Positive Emotions and the Meaning domains. Group interactions can evoke various emotions, but our findings indicate that co-creation discussions tend to generate positive emotions like joy, motivation, pride, gratitude, and reduced stress. The only study which reported learners’ frustration cited peers’ disengagement and an overwhelming experience. Nevertheless, most learners in this study enjoyed the collaborative process [44]. This scoping review expands on the benefits of co-creation, including an improved sense of meaning and purpose through enhanced empowerment, ownership, self-esteem, self-efficacy, self-awareness, self-confidence, and feelings of being helpful. Involving learners in the design process allowed them to build positive relationships where they reported feeling valued, respected, recognized, accepted, and appreciated. Literature suggests that acceptance and inclusion in a group create a greater sense of belonging that has been consistently associated with psychological well-being and positive psychosocial outcomes [60,61]. Research highlights that psychological safety is essential for enhancing the co-creation process [4]. This review added that co-creating with faculty can foster feelings of safety, delineating a ripple effect, largely due to the crucial role of team leaders in establishing a psychologically safe environment [62]. Therefore, despite the power dynamics, the involvement of staff/curricular designers with the learners can aid in creating a supportive environment that fosters psychological safety and change [63]. Finally, only a few studies reported learners’ sense of engagement and accomplishment during co-creation. The lack of reported engagement could be attributed to the limitation of studies to measure it effectively [64] and that accomplishing goals requires intentional decisions and actions, which is quite challenging to achieve in practice [65].

Studies involving small groups, new curriculum design, and true partnership have shown positive well-being outcomes across all five PERMA domains. Closer relationships in smaller groups likely contributed to these outcomes, while new curricula appeared to empower and excite learners. According to the CVTAE, people’s feelings about their achievements depend on their perceived control and the importance of those outcomes [21,22]. This could explain the value of empowering learners during the true partnership in designing their curricula, making them feel in control, eliciting positive emotions and establishing a positive relationship, finding meaning, engagement, and a sense of goal accomplishment. Although studies demonstrating pseudo-partnerships also reported positive well-being outcomes, six out of the seven involved learners in designing mental health initiatives, while only one study focused on curriculum development in long-term care. This might suggest that the reported well-being outcomes could have resulted from the content of these initiatives rather than the co-creation process itself.

### Implications and future directions

The review’s findings underscore the potential influence of co-creation on students’ well-being. A co-creation approach allows HPE learners to collaborate with staff in various contexts. This can include developing or modifying the curriculum as well as extracurricular activities. We encourage academic leaders to create environments where meaningful partnerships between learners and faculty/curriculum designers ultimately enhance learners’ well-being.

Although positive emotions were noted, they frequently appeared as secondary results rather than the primary focus of co-creation. Factors contributing to these outcomes remain unclear, highlighting the need for further investigation into the methods of operationalizing co-creation [66] across different HPEs. The included studies evaluated only the perceptions of the learners engaged in the co-creation process. It would be beneficial to explore the perceptions of those who received the co-created curriculum but were not directly involved to see if they had similar positive outcomes. Moreover, our results indicate that the positive outcomes observed in pseudo-partnership studies might be due to their focus on developing mental health initiatives. This scoping review focused on the reported outcomes related to learners’ well-being rather than evaluating the quality of the co-creation methods. A comparative study of outcomes between true and pseudo-partnerships might be helpful. We also suggest that future studies delve into how co-creation improves learners’ well-being. Lessons from such studies might help improve the quality of the co-creation processes and outcomes.

### Limitations

Our review has some limitations. Although we used broad search terms, we may have missed studies that involved learners in the design process but did not use these specific search terms. This could have led to the omission of relevant articles. Additionally, despite including all possible terminologies related to both positive and negative well-being outcomes in our search strategy, we might have overlooked studies that used different well-being-related terms, thus limiting our understanding of the full range of co-creation influences on learners’ well-being. Our search strategy was limited to English-language papers, so we might have missed non-English articles. Lastly, while we used the partnership process outcome model to examine the types of partnerships, we might have misinterpreted the nature of the partnerships due to some studies’ lack of clear descriptions of the co-creation process.

## Conclusion

The scoping review suggests that involving learners as co-creators in the design of their education can improve their well-being, as we found positive outcomes across all five domains of the PERMA model of well-being and happiness. We mainly noted these positive outcomes in those studies that involved small groups in co-creation, focused on designing new curricula, and used a true student-staff partnership approach. These findings present promising evidence that co-creation supports learners’ well-being, indicating a new direction for future research. Furthermore, the findings of this scoping review might encourage academic leaders to create opportunities and initiatives for involving learners in the design of their education.

## Additional File

The additional file for this article can be found as follows:

10.5334/pme.1726.s1 Supplement 2. Supplementary Table 1.
