# Supplementary material for: Fueling Inner Resources Through Co-Creation: A Scoping Review on the Impact of Co-Creation of Education on Learners’ Well-Being
Source: Perspect Med Educ. 2025 Mar 25;14(1):129–40. doi: 10.5334/pme.1726 (PMC11951960; doi:10.5334/pme.1726)
Supplement: Supplement 2. — Supplementary Table 1. [file pme-14-1-1726-s1.pdf]

Supplement 2

Summary of data from all included studies. Data charting included: (1) the main characteristics of the included studies, such as country and publication year, the article title, name of the author (s), journal name, and the methodology used (qualitative, quantitative, mixed), (2) the co-creation process: terms used to describe co-creation, co-creation setting, the number, training level and the professions of the learners involved in co-creation, and the level of partnership (i.e., true or pseudo-partnership), (3) the focus of co-creation, and (4) the reported mental health outcomes.

	**Study characteristics**					**The co-creation process**						**Focus of co-creation**	**Mental Health outcomes**
	**Country, Year of publication**	**Article title**	**Author (s) name**	**Name of the journal**	**Research method**	**Terms used to describe co-creation**	**Co-creation setting**	**Training level of learners**	**Professions of learners**	**No. of Participants per group**	**Level of Partnership**	**Focus of co-creation**	**Reported mental health outcome**
1	UK, 2023	Adaptation of Activate Your Wellbeing, a Digital Health and Well-being Program for Young Persons: Co-design Approach	Menna Brown; Emily Lord; Ann John.	JMIR Formative Research	Mixed methods	Co-design	Co-design workshops	Undergraduate	Medicine	4	Pseudo-partnership	Health and Wellbeing website	Enjoyable because of Friendly experience
2	Denmark, 2023	Embedding Scientific Communication and Digital Capabilities in the Undergraduate Biomedical Science Curriculum	Beverley C. Millar, Andrei Tarasov, Nigel Ternan, John E. Moore and Colette Murphy.	British Journal of Biomedical Science	Mixed methods	Co-design	Reflective focus groups	Undergraduate	Biomedical students	3	True partnership	Scientific communication and digital capabilities resource	Increased motivation, better engagement, better ownership, self-awareness, self-efficacy, self-confidence sense of pride, sense of accomplishment, and joy because of the creativity.
3	Australia, 2022	Promoting medical student engagement through co-development and peer-assisted learning: a new patient safety course as a case	Jesper Dybdal Kayser, Anne Mielke-Christensen, Doris Østergaard and Peter Dieckmann.	Advances in Simulation	Mixed methods	Co-developer	Group meetings	Undergraduate	Medicine	4	True partnership	Patient Safety course	Feeling of safety and safe environment. Being at the same place in the hierarchy, making refection possible and enables

		study											discussing communication on a more emotional level, where all ideas and thoughts are permitted.
4	Netherlands, 2020	Development of the Uni Virtual Clinic: an online programme for improving the mental health of university students	Louise M. Farrer, Amelia Gulliver, Natasha Katruss, Kylie Bennett, Anthony Bennett, Kathina Ali, Kathleen M. Griffiths	British Journal of Guidance & Counselling	Qualitative survey + interviews	Participation related terms	Focus groups	Undergraduate	Medicine	5	Pseudo-partnership	Mental Health Virtual Clinic program	Feeling of being valued,<br>Feeling of being respected.
5	Netherlands, 2019	Student participation in governance of medical and veterinary education: experiences and perspectives of student representatives and program directors	Stephanie NE Meeuwissen, Annemarie Spruijt, Jeroen W van Veen, Anton FPM de Goeij	Advances in Health Sciences Education	Mixed methods	Partnership related terms	Focus groups	Undergraduate	Mixed (medicine and Veterinary)	6	True partnership	Participation in institutional governance	Sense of respect,<br>self-confidence<br>sense of motivation<br>sense of gratitude<br>feeling of being valued
6	USA, 2020	Mind the gap: Teachers' conceptions of student-staff partnership and its potential to enhance educational quality	Samantha E Martens, Ineke HAP Wolfhagen, Jill RD Whittingham, Diana HJ M Dolmans	Medical Teacher	Mixed methods	Partnership related terms	Meetings	Undergraduate	Mixed (Medicine, Health Sciences, Biomedical Sciences And European Public Health.)	14	True partnership	Participation in PBL evaluation and reform	Sense of motivation
7	Norway, 2023	Development and implementation of a virtual "collaboratory" to foster interprofessional team-based learning using a novel faculty-student	Allison Shorten, David Alexandre Cruz Walma, Peter Bosworth, Brett Shorten, Bright Chang,	Journal of Professional Nursing	Qualitative	Partnership related terms	Sessions and Meetings	Undergraduate	Mixed (dentistry, nursing, occupational therapy, public health and social work)	16	True partnership	Interprofessional team-based learning course	Sense of enjoyment

		partnership	Matthew D. Moore, Laura Vogtle, Penni I. Watts										
8	France, 2023	Aiming for inclusion: processes taking place in co-creation involving students with disabilities in higher education	Anita Blakstad Bjørnerås, Eli Langørgen, Aud Elisabeth Witsø, Lisbeth Kvam, Ann-Elén Leithaug, Sissel Horghagen	International Journal of Inclusive Education	Mixed methods	Co-creation	Co-creation sessions	Undergraduate	Mixed (Social and Educational Sciences, Information Technology, Electrical Engineering, Medicine and Health Sciences, Engineering, Natural Sciences)	12	True partner ship	An intervention to meet the goals of inclusive education	Feeling recognized and accepted because of expressing feelings and thoughts, articulating frustrations, and being acknowledged.
9	Qatar, 2021	Tutor–Student Partnership in Practice OSCE to Enhance Medical Education	Eve Cosker, Valentin Favier, Patrice Gallet, Francis Raphael, Emmanuelle Moussier, Louise Tyvaert, Marc Braun, Eva Feigerlova.	Medical Science Educator	Qualitative	Co-creation	Training sessions	Undergraduate	Medicine	5	True partner ship	A practice OSCE	Joy
10	Australia, 2023	Sailing the boat together: Co-creation of a model for learning during transition	Shireen Suliman, Karen D. Konings, Margaret Allen, Ayad Al-Moslih, Alison Carr	Medical Teacher	Qualitative	Co-creation	Co-creation sessions	Undergraduate	Medicine	6	True partner ship	Transition to Residency Model	Reduced stress
11	UK, 2010	Expanded partnerships between medical faculty and medical students: Developing a Global Health curriculum as an example of ‘student-led	Nancy Merridew & David Wilkinson	Medical Teacher	Qualitative	Student's voice	Design workshops	Undergraduate	Medicine	20	True partner ship	A Global Health curriculum.	Joy

		learning' at the University of Queensland, Australia											
12	Canada, 2023	Developing inductions to support mental health and wellbeing in doctoral researchers: findings from a qualitative co-design study with doctoral researchers and university stakeholders	Patricia C Jackman, Rebecca Sanderson, Lisa Jacobs	European Journal of Higher Education	Qualitative	Co-production	Focus groups	Postgraduate	Mixed (social sciences, medical sciences, science, technology, engineering, and mathematics (STEM), and arts and humanities)	5	True partnership	Inductions to support mental health and wellbeing in doctoral researchers.	Feeling positive about their involvement. Sense of enjoyment, Sense of confidence, Sense of acceptance, Sense of being valued.
13	Across continents, 2023	Whom do we include and when? Participatory design with vulnerable groups	Elise Hodson, Annukka Svanda, Nastaran Dadashi	CoDesign International Journal of CoCreation in Design and the Arts	Mixed methods	Participation related terms	Planning sessions	Undergraduate	Nursing	5	True partnership	Understanding of challenges faced by vulnerable nursing student	Confidence to come forward.
14	Australia, 2023	Partners in academic endeavour: Characterising student engagement across internationally excellent medical schools	Flávia Freitas, Kathleen E Leedham-Green, Susan F Smith, Manuel João Costa	Medical Teacher	Qualitative	Engagement related terms	Multiple case study design	Undergraduate	Medicine	9	True partnership	Understanding institutional excellence in student engagement	Confidence, motivation
15	UK, 2017	Student as partners: supporting work ready graduates through co-creation	Katrina Plastow, Sophia Karanicolas, Catherine Snelling, Rosie Cooper	9th International Conference On Education And New Learning Technologies (Edulearn17)	Mixed methods	Co-creation	Design workshops	Undergraduate	Dentistry	6	True partnership	Stainless Steel Crowns (SSCs) and Exodontia Induction modules	Joy
16	France, 2023	Co-creation to Develop Interventions to Facilitate Deep Reflection for Dental Students	Faith Campbell, Nicole Hassoon, Khalil Jiwa, Julia Ridsdill-Smith,	Perspectives on Medical Education	Qualitative	Co-creation	Co-creation workshops	Undergraduate	Dentistry	5	True partnership	A comprehensive intervention to facilitate deep reflection for undergraduate dental students	Confidence and motivation because demonstrate the value that they found in both co-creation and reflective practice.

			Amie Smith, Helen Wilson, Kirsten Jack, Helen Rogers										
17	Netherlands , 2021	Teaching bacterial infections in pharmaceutical studies: why not 'with students' instead of 'to students'?	Matthieu Eveillard, Isabelle Baglin, Samuel Legeay	Fems Microbiolog y Letters	Qualitative	Cco-creation	Sessions	Undergraduate	Pharmacy	4	True partner ship	Clinical Bacteriology	Self-confidence and motivation because they worked with teachers
18	US, 2022	What is it like to organize a large-scale educational event for fellow students? A qualitative exploration of student participation in curriculum design	Gert Olthuis, Florieke Eggermont, Bas Schouwenberg , Anke Oerlemans, Esther Tanck	B Medical Education	Qualitative	Participati on related terms	Meetings	Undergraduate	Mixed (biomedical sciences/ Medicine)	9	True partner ship	The Radboud Student Conference (RSC)	Joy because teachers were approachable.
19	US, 2023	A participatory study of college students' mental health during the first year of the COVID-19 pandemic	Chulwoo Park, Melissa McClure Fuller, Thea Marie Echevarria, Miranda Worthen	Frontiers in Public Health	Qualitative	Participati on related terms	Groups	Undergraduate	Population Health	5	Pseudo - partner ship	A Novel Participatory Approach to a Course-based Undergraduate Research Experience (CURE)	Enjoyed how the curriculum changed to reflect that semester's cohort.
20	New Zealand, 2014	Continuous curricular feedback: a formative evaluation approach to curricular improvement.	Stanley Goldfarb, Gail Morrison	Academic Medicine	Participation in curriculum design"	Involvem ent related terms	Evaluation Sessions	Undergraduate	Medicine	10	True partner ship	Curriculum evaluation model	Enjoy
21	Canada, 2017	Co-creation Improves Pre-exam Motivation and Self-Efficacy for Medical Students	Diane Kenwright, Wei Dai, Bonnie White, Joshua Smith, Shaun Collings, Rebecca Grainger	Medical Science Educator	Qualitative (Participatory approach)	Co-creation	Groups	Undergraduate	Medicine	7	True partner ship	Pathology Revision course	High levels of autonomy, improved levels of confidence and motivation, and self-efficacy because suited their learning needs
22	Belgium, 2017	Exploring dental student participation	Jeremy Huynh, Leeann R	Gerodontolo gy	Mixed methods	Participati on related	Focus group	Undergraduate	Dentistry	9	Pseudo -	Interdisciplinar y care	Confidence because to get that

		in interdisciplinary care team conferences in long-term care.	Donnelly, Mario A Brondani			terms					partner ship	conference	experience, to speak up, to bring their professional skill set to the table and to try it out…when they do speak up they have lots of positive things to contribute to the conversation.
23	UK, 2023	Accessibility, Retention and Interactivity of Online Co-Creation Workshops: A Qualitative Post-Hoc Analysis	Anke Boone, Lutgart Braeckman, Nele Michels, Hanne Kindermans, Elke Van Hoof, Van den Kris Broeck, Lode Godderis	International Journal of Qualitative Methods	Qualitative (A participatory action research)	Co-creation	Co-creation workshops	Undergraduate	Medicine	19	True partner ship	Investigate the online format of co-creation workshops	Enjoy Psychological safety and Freedom of expression
24	UK, 2019	The benefits of student-led health promotion intervention	Athene Lane-Martin	The Journal of Mental Health Training, Education and Practice	Qualitative	Student-led	Group	Undergraduate	Population Health	3	Pseudo - partner ship	A health promotion intervention	Raised self-awareness, enthusiasm confidence, enjoyment autonomy because they were able to reflect on their own skills and attributes
